# Supplementary material for: Biomimetic Synthesis of PANI/Graphitic Oxidized Carbon Nitride for Supercapacitor Applications
Source: Polymers (Basel). 2022 Sep 19;14(18):3913. doi: 10.3390/polym14183913 (PMC9503369; doi:10.3390/polym14183913)
Supplement: Supplementary file 1 [file polymers-14-03913-s001.zip › polymers-1896309-supplementary.pdf]

Supplementary Materials

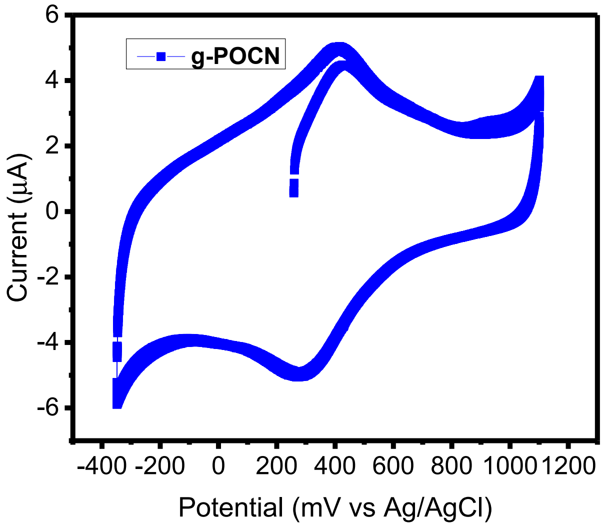

Figure S1. Cyclic voltammetry g-POCN in 0.5 M H_2SO_4 at 100 mV/s (working potential −0.35 V to 1 V).

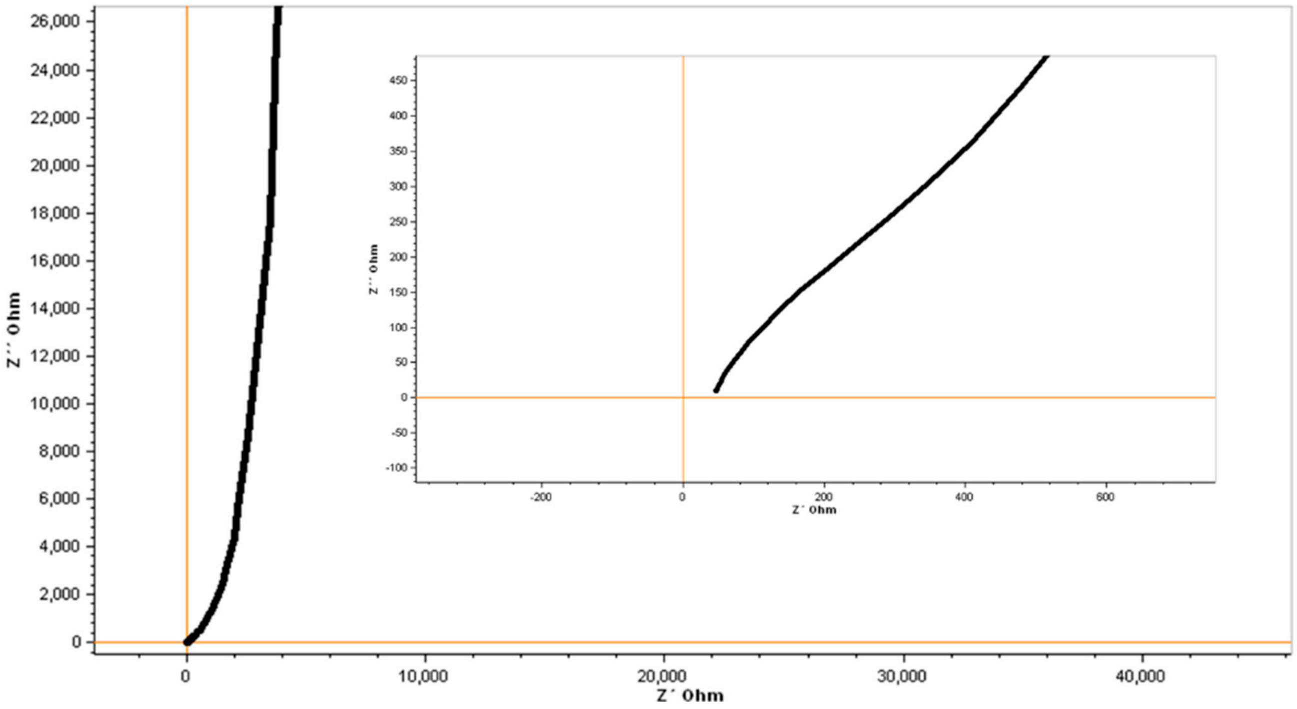

Figure S2. EIS of g-POCN in 0.5 M H_2SO_4 obtained at scan interval from 100 mHz to 100 kHz with amplitude of 10 mV VA to Eoc.

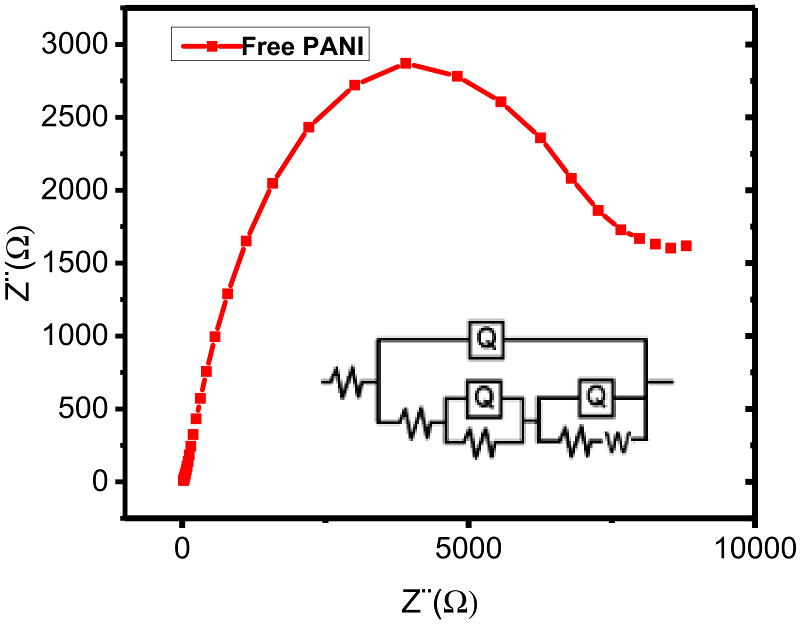

Figure S3. Nyquist diagram of PANI in 0.5 M H_2SO_4 obtained at scan interval from 100 mHZ to 100 kHz with amplitude of 10 mV VA to Eoc.

Table S1. Relevant studies recently reported and their comparison with the present work.

Material	Method of synthesis	Electrolyte	Potential window (V)	Specific capacitance F/g (at current density, A/g, scan rate)	-Capacity retention -Cycle number -Current density (A/g)	Year	Ref
CoFe-LDH/PANI	Hydrothermal synthesis	KOH 6M	−0.1 to 0.7	1686 (1)	98% 10000 20	2022	[1]
PANI/CNT/ Elastic polymer fiber	Electro-polymerization	PVA/ H_3PO_4	0.0 to 1	111.6 (0.5)	90% 2000 1	2014	[2]
PANI/CNT/ Elastic cord	Electro-polymerization	PVA/ H_2SO_4	−0.2 to 0.6	394	75.5% 12000 30	2018	[3]
PANI-MWCNT-Ni(OH)₂	Chemical precipitation	KOH 1 M	0.0 to 0.49	1013 (1)	75% 1000 25	2020	[4]
PANI@Co-Porphyrins	/	H_2SO_4 1 M	0 to 0.6	823 (0.5)	91% 1000 5	2019	[5]
GO/PANI/ CNT-SS	/	KCl 1 M	1.0 to 1.6	331.49 (5 mV/s)	83% 5000 /	2019	[6]
PANI/ PEDOT/ PANI/	/	PVA/ H_2SO_4	0.2 to −0.4	61 F/cm^3 (1 A/cm^3)	73% 1000 /	2019	[7]

UrGO//<br>PEDOT-MoS_2							
PANI/GO/<br>PDMS	Micro-emul-<br>sion *in-situ*<br>polymerization	LiCl<br>1 M	− 0.2 to 1	576 (1)	/<br>10000<br>5	2018	[8]
$BiVO_4$/<br>PANI	*in situ* chemical<br>oxidative<br>polymerization	KOH<br>1 M	0 to 0.66	701 (1)	95.4%<br>5000<br>1	2020	[9]
co-MOF/<br>PANI	*in situ* chemical<br>oxidative<br>polymerization	KOH<br>1 M	0 to 0.62 V	504 (1)	90%<br>5000<br>2	2020	[10]
CeO_2/<br>PANI	/	HCl<br>1 M	0 to 0.8 V	1452 (2)	~0%<br>1500<br>20	2018	[11]
PANI/<br>g-POCN	Biomimetic<br>Synthesis	H_2SO_4<br>0.5 M	−0.2 to 0.9	450.5 (0.2)	80<br>500<br>3	2022	This<br>work

References

1. Mahmood, A.; Zhao, B.; Javed, M.S.; He, D.; Cheong, W.; Han, D.; Niu, L. Unprecedented Dual Role of Polyaniline for Enhanced Pseudocapacitance of Cobalt–Iron Layered Double Hydroxide. *Macromol. Rapid Commun.* **2022**, *43*, 2100905. https://doi.org/10.1002/marc.202100905.
2. Zhang, Z.; Deng, J.; Li, X.; Yang, Z.; He, S.; Chen, X.; Guan, G.; Ren, J.; Peng, H. Superelastic Supercapacitors with High Performances during Stretching. *Adv. Mater.* **2015**, *27*, 356–362. https://doi.org/10.1002/adma.201404573.
3. Ren, D.; Dong, L.; Wang, J.; Ma, X.; Xu, C.; Kang, F. Facile Preparation of High-Performance Stretchable Fiber-Like Electrodes and Supercapacitors. *ChemistrySelect* **2018**, *3*, 4179–4184. https://doi.org/10.1002/SLCT.201702725.
4. Thomas, L.; Pete, S.; Chaitra, K.; Venkatesh, K.; Gopalkrishna, B.; Nagaraju, K. Facile synthesis of PANI-MWCNT-$Ni(OH)_2$ ternary composites and study of their performance as electrode material for supercapacitors. *Diam. Relat. Mater.* **2020**, *106*, 107853. https://doi.org/10.1016/J.DIAMOND.2020.107853.
5. Deyab, M.A.; Mele, G. PANI@Co-Porphyrins composite for the construction of supercapacitors. *J. Energy Storage* **2019**, *26*, 101013. https://doi.org/10.1016/J.EST.2019.101013.
6. Dasdevan, N.; Abdah, M.A.A.M.; Sulaiman, Y. Facile Electrodeposition of Poly(3,4-ethylenedioxythiophene) on Poly(vinyl alcohol) Nanofibers as the Positive Electrode for High-Performance Asymmetric Supercapacitor. *Energies 2019, Vol. 12, Page 3382* **2019**, *12*, 3382. https://doi.org/10.3390/EN12173382.
7. Khoh, W.H.; Wee, B.H.; Hong, J.D. High performance flexible solid-state asymmetric supercapacitor composed of a polyaniline/PEDOT/polyaniline/ultralarge reduced graphene oxide tetralayer film and a PEDOT/MoS2 composite film. *Colloids Surfaces A Physicochem. Eng. Asp.* **2019**, *581*, 123815. https://doi.org/10.1016/J.COLSURFA.2019.123815.
8. Li, K.; Huang, Y.; Liu, J.; Sarfraz, M.; Agboola, P.O.; Shakir, I.; Xu, Y. A three-dimensional graphene framework-enabled high-performance stretchable asymmetric supercapacitor. *J. Mater. Chem. A* **2018**, *6*, 1802–1808. https://doi.org/10.1039/C7TA09041B.
9. Srinivasan, R.; Elaiyappillai, E.; Anandaraj, S.; Duvaragan, B. kumar; Johnson, P.M. Study on the electrochemical behavior of BiVO4/PANI composite as a high performance supercapacitor material with excellent cyclic stability. *J. Electroanal. Chem.* **2020**, *861*, 113972. https://doi.org/10.1016/J.JELECHEM.2020.113972.
10. Srinivasan, R.; Elaiyappillai, E.; Nixon, E.J.; Sharmila Lydia, I.; Johnson, P.M. Enhanced electrochemical behaviour of Co-MOF/PANI composite electrode for supercapacitors. *Inorganica Chim. Acta* **2020**, *502*, 119393. https://doi.org/10.1016/J.ICA.2019.119393.
11. Nallappan, M.; Gopalan, M. Fabrication of CeO2/PANI composites for high energy density supercapacitors. *Mater. Res. Bull.* **2018**, *106*, 357–364. https://doi.org/10.1016/J.MATERRESBULL.2018.05.025.
